# Comparison of Bacterial Adhesion on Two Different Suture Materials After Tooth Extraction in Women Receiving Antiresorptive Therapy: An Exploratory Clinical Study with Prospective Data Collection

**DOI:** 10.3390/jcm15072737

**Published:** 2026-04-04

**Authors:** Anna Mölzer, Jesika Kotorri, Lotta Gath, Jakob Fehlhofer, Marco Rainer Kesting, Christian Bogdan, Roman G. Gerlach, Mayte Buchbender

**Affiliations:** 1 Department of Oral and Maxillofacial Surgery, Universitätsklinikum Erlangen, Friedrich-Alexander-Universität (FAU) Erlangen-Nürnberg, Glückstrasse 11, 91054 Erlangen, Germany; anna.moelzer@fau.de (A.M.); lottagath@hotmail.de (L.G.); fehlhofer.jakob@gmail.com (J.F.); marco.kesting@uk-erlangen.de (M.R.K.); 2 Mikrobiologisches Institut—Klinische Mikrobiologie, Immunologie und Hygiene, Universitätsklinikum Erlangen, Friedrich-Alexander-Universität (FAU) Erlangen-Nürnberg, 91054 Erlangen, Germany; jesika.kotorri@medisin.uio.no (J.K.); christian.bogdan@uk-erlangen.de (C.B.); roman.gerlach@uk-erlangen.de (R.G.G.)

**Keywords:** antiresorptive therapy, bacterial load, MRONJ, suture material, tooth extraction

## Abstract

**Background/Objectives:** Medication-related osteonecrosis of the jaw (MRONJ) is a rare but severe complication of antiresorptive therapy for osteoporosis. This study investigated bacterial adhesion and microbial composition on two suture materials and their potential impact on early wound healing following tooth extraction in patients receiving antiresorptive therapy. **Methods:** In this prospective exploratory clinical study with partially randomized allocation, female patients undergoing antiresorptive therapy were evaluated for clinical parameters, including the Mombelli Plaque Index (MPI), Mombelli Bleeding Index (MBI), oral smear analysis, and Early Wound Healing Score (EHS). Suture samples (*Vicryl* and *Monocryl, Ethicon*, Germany) were removed after 10 days, measured, and weighed. Bacterial DNA was isolated and quantified by qPCR targeting the albumin and 16S rRNA genes. In addition, 16S rRNA gene amplicon sequencing was performed to assess the microbial community composition. Statistical and bioinformatic analyses were conducted to compare materials and evaluate the clustering patterns. **Results:** Fifty-two suture samples were analyzed. *Vicryl* exhibited significantly higher 16S rRNA gene copy numbers than *Monocryl*, indicating increased bacterial colonization, whereas albumin gene copy numbers were significantly higher in *Monocryl*. The suture weight correlated primarily with albumin gene copy numbers. Amplicon sequencing revealed no material-dependent differences in the microbial composition; instead, samples clustered predominantly by patient, particularly in split-mouth cases. The wound healing outcomes based on the EHS were comparable between materials. **Conclusions:** Although *Vicryl* and *Monocryl* differ in bacterial load and host material deposition, the microbial community composition is primarily patient-specific and the clinical healing outcomes are similar. Surgical management and patient-related factors appear more critical than suture material selection.

## 1. Introduction

Medication-related osteonecrosis of the jaw (MRONJ) is characterized by exposed or probeable bone that persists for more than eight weeks because of denture-induced pressure, periodontitis, or, most significantly, invasive dentoalveolar procedures such as tooth extraction. Patients with these symptoms have usually undergone therapy with antiresorptive or antiangiogenic drugs, but have not received radiotherapy of the head and neck region [1,2,3]. In medicine, antiresorptive agents are employed to treat osteoporosis, as these compounds increase bone density and thereby reduce the risk of bone fractures. Three different classes of antiresorptive medications can be distinguished. Bisphosphonates induce the apoptosis of osteoclasts which are responsible for bone resorption. These include drugs such as alendronic acid, risedronic acid, ibandronic acid, and zoledronic acid. Denosumab, a human monoclonal antibody able to block the receptor activator of nuclear factor κ-B ligand (RANKL), and raloxifene, which is a selective estrogen receptor modulator (SERM), interfere with bone remodeling processes by preventing the recruitment of osteoclasts. While all of these compounds are used for the treatment of osteoporosis, the latter is also applied as a preventive measure [4].

In patients receiving antiresorptive therapy, the jawbone and gingival epithelium can no longer regenerate properly, as the drugs impair bone remodeling due to the loss of osteoclasts and inhibit angiogenesis. This promotes the development of either nitrogen-containing bisphosphonate-related osteonecrosis of the jaw (BRONJ), which was first described in 2003 [5]. Clinically, this manifests as open, nonhealing bone defects in the jawbone and reduced regeneration of soft tissue in the oral cavity [6]. In 2010, the occurrence of osteonecrosis of the jaw (ONJ) in connection with denosumab was also described for the first time, which is why the term ARONJ, i.e., antiresorptive-related osteonecrosis of the jaw, was coined a year later. Since antiangiogenic drugs can also cause this type of necrosis, MRONJ serves as an overarching term [7].

Although the likelihood of developing MRONJ as an osteoporotic woman undergoing low-dose treatment with bisphosphonates is very low (0.001% to 0.15%) [1], certain rules should be followed to prevent this pathology [1,3]. These are described in official guidelines for preventing the antiresorptive-related osteonecrosis of the jaw, e.g., peri-operative antibiotic treatment, atraumatic operation, the smoothing of bone edges during surgery, and the primary closure of the extraction socket with a mucoperiosteal flap [3,8]. Accordingly, there are recommendations regarding the surgical procedure, but no reference has been made to the type of suture material. While MRONJ represents a clinically relevant complication, the present study does not directly investigate MRONJ occurrence but rather focuses on surrogate parameters such as bacterial adhesion and early wound healing.

Several studies have shown that there are significant differences in bacterial adhesion depending on the type of suture material used. The results of these studies support the conclusion that, compared with multifilament suture material, monofilament suture material is associated with lower bacterial loads [9,10,11,12,13,14,15,16,17,18,19,20,21,22,23]. In addition, it was observed that monofilament suture material led to fewer tissue reactions and was associated with enhanced wound healing compared with multifilament suture material [9,10]. Although these clinical studies focused on dentoalveolar procedures, there is currently little research on the bacterial load on suture material in patients receiving antiresorptive therapy.

In our department, tooth extractions of patients who receive antiresorptive agents follow a standardized protocol. With respect to primary wound closure, all patients receive a rotational full flap with a mesial relieving incision and tension-free suturing. Standard multifilament suture material is routinely used, but, in this study, a pseudomonofilament suture material was used for comparison. We tested the hypothesis that monofilament sutures exhibit a reduced bacterial load as compared to braided sutures.

This exploratory prospective clinical study aimed to examine the wound healing process following oral surgery in patients receiving antiresorptive agents for the treatment of osteoporosis. Specifically, we investigated the potential differences in the bacterial loads between a monofilament and a multifilament absorbable suture material.

## 2. Materials and Methods

### 2.1. Study Design and Patient Recruitment

In the present prospective exploratory clinical study with partially randomized allocation, female patients who were diagnosed with postmenopausal osteoporosis and who were scheduled to undergo dentoalveolar tooth extraction were enrolled at the Department of Oral Maxillofacial Surgery of the University Hospital of Erlangen. The patients included were receiving antiresorptive drugs as treatment for osteoporosis. Subjects were enrolled and examined from August 2023 to October 2024. The study was approved by the ethics committee of the Medical Faculty of Friedrich-Alexander University of Erlangen-Nürnberg, Germany (approval no. 23-198-B) and executed in accordance with the guidelines of the Declaration of Helsinki. This study was also registered retrospectively as a non-interventional study in the German clinical trial registry (DRKS00037740). Due to the retrospective registration of the study, the investigation was not conducted under a fully predefined prospective protocol in the sense of a registered clinical trial. Instead, the study should be understood as an exploratory clinical investigation with predefined procedural standards but without a formally registered prospective analysis plan. Prior to inclusion in this study, all patients were informed and gave written consent. Initial examination and dentoalveolar tooth extraction surgery were performed on all of the subjects according to a standardized protocol, followed by further examination at day 3, 5, 7, and 10 after surgery. On the 10th day after surgery, suture samples were collected. Two different suture materials were applied in a controlled manner. The initial allocation of suture material was randomized, followed by an alternating sequence. In selected patients requiring multiple extractions, a split-mouth design was used to allow intra-individual comparison. Therefore, despite controlled allocation of suture materials, the study should be interpreted within an exploratory observational context rather than as a confirmatory interventional trial.

The exclusion criteria were as follows:

- Antiresorptive therapy for malignant disease;
- Radiotherapy around the head and neck region;
- Untreated diabetes mellitus;
- Immune disorders (e.g., HIV);
- Treatment with immunosuppressive agents.

### 2.2. Dental and Wound Healing Parameters and Suture Sample Collection

The examinations prior to and after surgery as well as the measurement of the samples were conducted by clinical practitioners of the Department of Oral Surgery and one examiner (A.M.). Parameters, surgeries, and sampling were always performed following a certain standardized protocol:

1. Modified Mombelli Plaque Index (MPI) assessment: The grades are from 0 to 3. Grade 0: no detection of plaque; Grade 1: plaque is not visible but is detectable by probing; Grade 2: visible plaque accumulation without probing; Grade 3: obvious plaque accumulation even on the tooth’s smooth surfaces.
2. Modified Mombelli Bleeding Index (MBI) assessment: The grades are from 0 to 3. Grade 0: no bleeding when the periodontal probe was inserted into the sulcus; Grade 1: visible and isolated bleeding points; Grade 2: bleeding points merged into a red line; Grade 3: heavy or profuse bleeding.
3. Smear of the oral cavity: The site of surgery can be used to examine potential contamination or infection with bacteria that affect wound healing. Smears were sent to and analyzed by the Institute of Clinical Microbiology, Immunology, and Hygiene of the University Hospital Erlangen.
4. Early Wound Healing Score (EHS): The EHS was used to evaluate early wound healing 10 days after surgery. Three issues, namely, clinical signs of hemostasis (CSH), clinical signs of inflammation (CSI), and clinical signs of re-epithelialization (CSR), were categorized. For the CSH and CSI, a maximum of 2 points can be achieved, whereas a full score for CSR results in 6 points. The parameters considered are bleeding and fibrin for CSH, the amount of redness and swelling along the incision length for CSI, and the condition of the incision margins (visible distance, in contact, or merged) for CSR, resulting in a maximum total score of 10 points.

Both types of suture material used for wound closure were products of *Ethicon*, Germany. *Vicryl 5-0*, a multifilament and braided suture, and *Monocryl 5-0*, a monofilament, single-strand suture, were compared. *Vicryl* is an absorbable suture material that consists of polyglactin 910, whereas *Monocryl* is composed of polyglecaprone 25 and categorized as a pseudomonofilament and absorbable suture material [24]. The initial allocation of suture material was randomized, followed by an alternating sequence to ensure balanced distribution.

To prevent a negative impact on wound healing by removing crucial fastening sutures, two additional single-button sutures were placed to the most mesial and distal points of the mucoperiostal mesial rotational flap during the wound closure at the end of the operation. For patients requiring extraction of multiple teeth in different quadrants of the jaw, a split-mouth design was applied. In these cases, both types of suture material were used within a single surgical procedure. Patients who underwent two separate surgeries were included in both sample collections. A split-mouth design was employed to minimize inter-individual variability and to allow for a direct within-patient comparison between the two suture materials. Patients received antibiotic therapy with amoxicillin (twice/day [1-0-1], 1000 mg each) from two days prior to 10 days after surgery. Suture samples were collected in 1.5 mL tubes 10 days postoperatively. Their length was measured with the help of a dermatoscope without sample contact. The weight of the sutures was determined with a precision scale after DRK. The tare weight of the tubes was obtained prior to sample collection. The sample tubes were kept frozen at −80 °C upon further processing.

### 2.3. Bacterial DNA Isolation and Quantification

Before isolation of the bacterial DNA, the samples were thawed at room temperature for 20 min and weighed on an AX105 Delta Range precision scale (Mettler Toledo, Gießen, Germany). Briefly, samples were incubated in a proteinase K-based digestion buffer and lysed using the Bead Ruptor 24 (Omni International, Kennesaw, GA, USA). DNA was extracted using a ZymoBIOMICS Quick-DNA Bacterial/Fungal Microprep Kit (Zymo Research, Freiburg, Germany) following the manufacturer’s protocol. DNA concentrations were subsequently quantified in a Qubit Flex fluorometer (Thermo Fisher Scientific, Dreieich, Germany).

### 2.4. Amplification of the 16S rRNA Gene

Amplification of the 16S rRNA gene V1–V3 region was carried out with 12 phased primer pairs as described by Seidel et al. [25] with the following PCR conditions: initial denaturation (95 °C, 3 min), 25 cycles of denaturation (95 °C, 30 s), annealing (55 °C, 30 s), and extension (72 °C, 30 s); final extension (72 °C, 5 min); and hold (4 °C, ∞). PCR products were purified using 0.8× volumes AMPure XP beads (Beckman Coulter Genomics, Brea, CA, USA). Nextera XT Index KIT v2 Set B and C primers (Illumina, San Diego, CA, USA) were used for adapter and index addition. The indexing PCR was carried out with eight cycles using the previously specified program. The PCR products were purified with 1.12× volumes AMPure XP beads. Amplicon libraries were sequenced using a 600-cycle v3 Reagent Kit on an Illumina MiSeq for paired-end sequencing.

### 2.5. qPCR for the Albumin Gene and 16S rRNA Gene

Bacterial abundances on the sutures were estimated by amplification of the 16S V3–V4 region in a qPCR as described before [26] in combination with the Luna Universal qPCR Mastermix (New England Biolabs, Frankfurt am Main, Germany) on a ViiA 7 real-time PCR system (Thermo Fisher Scientific). To estimate the 16S rRNA gene copy numbers, a standard curve based on dilutions of four artificial 1000 bp long 16S rRNA genes derived from Tourlousse et al. [27] (Ec5001, Ec5003, Ec5005, and Ga5505) was included. The qPCR was carried out using the following program: 95 °C, 15 min; followed by 45 cycles of 95 °C, 15 s; 50 °C, 20 s; and 72 °C, 30 s. Host-derived DNA was quantified by targeting the albumin gene in a qPCR as described previously [28], but using a modified probe VIC-AGCTCTGGAAGTCGAT-NFQ-MGB together with the FastStart Universal Probe Master (Roche, Basel, Switzerland) on a QuantStudio 5 (Thermo Fisher Scientific) and the following program: 50 °C, 2 min; 95 °C, 10 min; and 50 cycles of 95 °C, 15 s; and 60 °C, 1 min. For absolute quantification, a standard curve based on dilutions of a plasmid containing the human albumin gene was included.

### 2.6. Primary and Secondary Outcomes

The primary outcome was the comparison of the amount of bacteria adhering to the two different suture materials. The secondary endpoint was the correlation between the EHS and the clinical parameters and the bacterial adhesion to the suture material. In addition, the relationship between the bacterial colonization prior to surgery (preoperative smear) and the bacterial colonization of the suture material was analyzed.

### 2.7. Computational and Statistical Analysis

Sequence data were demultiplexed with the “Generate Fastq” workflow in Illumina MiSeq Reporter. Raw read data are available through NCBI BioProject accession number PRJNA1354425. Primer and adapter sequences and reads with N > 0 and expected error (EE) > 5 were removed with Cutadapt v1.6. Read merging and clustering into zero-radius operational taxonomic units (ZOTUs) was performed using the VSEARCH algorithm [29]. Chimera detection was done with uchime3_denovo. Non-target and artifact sequences were filtered out using SortMeRNA 4.3.6 with SILVA 138.1 as reference database. Taxonomic classification of ZOTU sequences was performed using SINA v1.7.2 [30] with SILVA database version 138.2 and BLAST v2.15.0 [31] using the databases refseq_rna (accessed July 2024), ref_prok_rep_genomes (accessed June 2024), and nt_prok (accessed July 2024). Further analysis was performed using custom scripts in R v4.3.1. Alpha diversity was calculated using Shannon’s and Simpson’s diversity index on ZOTU counts following the filtering out of low-abundance ZOTUs (≤0.05%) and contaminants using the Phyloseq package [32]. A Wilcoxon rank-sum test and a *t*-test were used to assess the statistical significance of diversity. Beta-diversity was summarized using a principal coordinate analysis (PCoA) on Bray–Curtis dissimilarities of log1p-transformed ZOTU counts. Differences in suture weights and lengths were assessed using a Wilcoxon rank-sum test. Normal distribution of samples was determined using a Shapiro–Wilk test. Correlation analysis between suture characteristics was performed using Pearson’s correlation. Statistical analyses were performed at the sample level, with each suture sample treated as an independent observation.

## 3. Results

### 3.1. General Patient Data

A total of 21 female patients, who had previously received or were currently taking antiresorptive agents, were included in the present study; 52 suture samples were obtained from these patients: 30 *Vicryl* sutures and 22 *Monocryl* sutures. We implemented a split-mouth design in four patients (PT6, PT8, PT9, and PT13) in whom multiple teeth in different quadrants of the oral cavity were extracted. During these surgeries, *Vicryl* and *Monocryl* were used to compare both suture types in one patient. One woman (PT15) underwent two surgical procedures at different times; thus, she participated in the sample collection of both suture materials. Two suture samples were collected for each extraction field, resulting in a total of 52 samples. The detailed patient data are presented in Table 1.

### 3.2. Primary Outcomes

**Figure 1:**
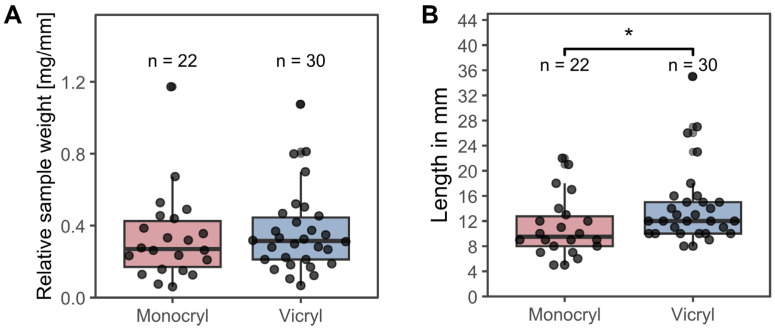
(**A**) Relative suture weights in milligrams per millimeters. (**B**) Suture lengths in millimeters. The boxes represent the 1st and 3rd quartile of the obtained data and show the median as horizontal line. Whiskers represent the 1.5× interquartile range. * equals *p* ≤ 0.05.

**Figure 2:**
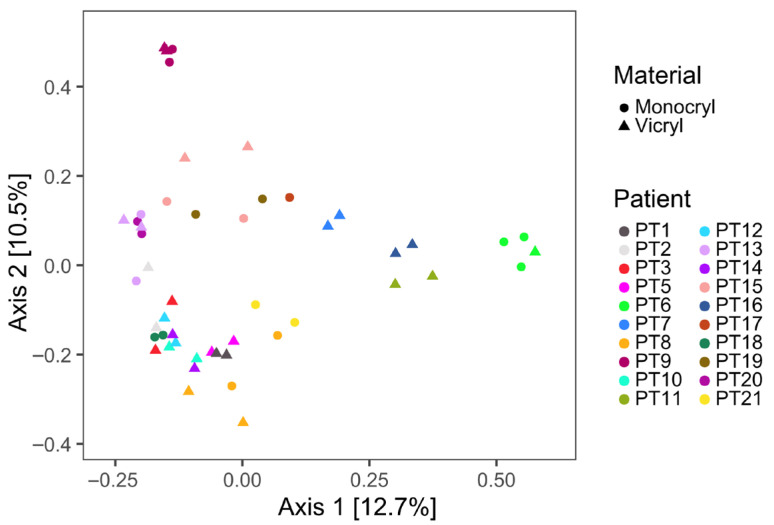
PCoA of Bray–Curtis Dissimilarities of log10p-transformed ZOTU counts. Samples are colored according to the source patient and the different suture material is indicated by the point shape. Surgeries using split mouth design: PT6, PT8, PT9, and PT13; same patient, two surgeries: PT15.

**Figure 3:**
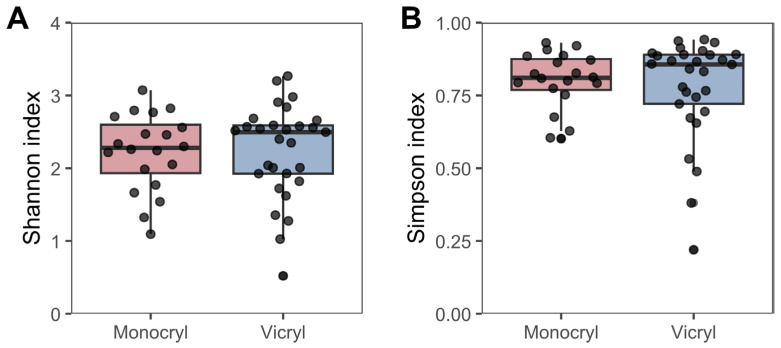
(**A**) Shannon and (**B**) Simpson alpha diversity measures based on suture material are shown. No significant differences in alpha diversity between *Monocryl* and *Vicryl* samples were observed in either index. Boxes show 1st and 3rd quartile and median.

**Figure 4:**
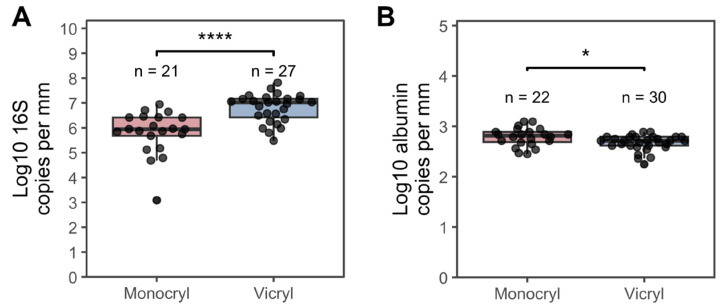
Estimation of bacterial load and host cell attachment of oral suture material via qPCR. (**A**) 16S rRNA gene copy numbers normalized to sample length. (**B**) Albumin gene copy numbers normalized to sample length. Boxes show 1st and 3rd quartile and median and whiskers represent the 1.5× interquartile range. * equals *p* ≤ 0.05, **** equals *p* ≤ 0.0001.

**Figure 5:**
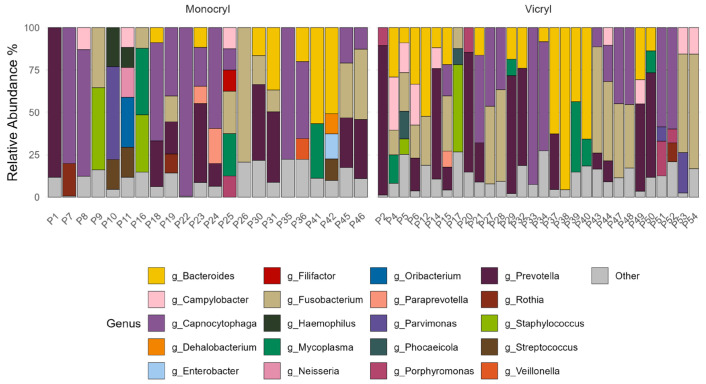
Relative abundance of bacterial genera on both suture materials, listed by suture ID. Genera comprising less than 8% for the total composition were grouped as “Other”.

**Figure 6:**
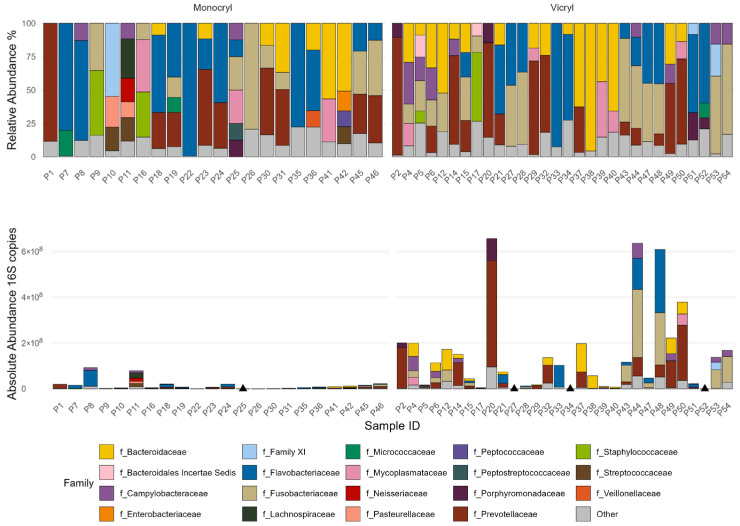
Relative and absolute abundance of taxonomic families, listed by suture ID. Families with a relative abundance of <8% were grouped as “Other”. Triangles indicate samples with no 16S rRNA genes amplified by qPCR.

**Figure 7:**
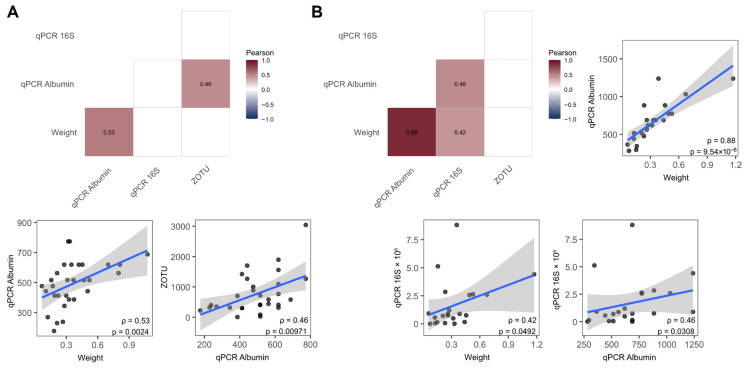
Pearson correlation between length-normalized sample weight (Weight), length-normalized albumin (qPCR Albumin) and 16S (qPCR 16S) gene copy numbers, and length-normalized ZOTU read counts. (**A**) A matrix of significant correlations (*p* < 0.05) for *Vicryl* samples is depicted. Pairwise bivariate scatter plots with a linear trendline, confidence interval bands, Pearson correlation coefficient (ρ), and *p*-value are shown for significant correlations. (**B**) Similar data as shown in (**A**) is depicted for *Monocryl* samples.

Table 2 presents the data that were utilized for further computational and statistical analysis, which are shown in Figure 1, Figure 2, Figure 3, Figure 4, Figure 5, Figure 6 and Figure 7. This includes variables such as the suture length, weight, and 16S rRNA gene and albumin gene copy number, in addition to the patient and suture IDs.

#### 3.2.1. Suture Weights and Lengths

To assess whether the suture material influenced the sample weight, we first determined the dry weight per millimeter for each material. The dry weight was 0.32 ± 0.0088 mg/mm for *Monocryl* and 0.27 ± 0.0058 mg/mm for *Vicryl*. The relative sample weight per millimeter was then calculated by subtracting the respective suture dry weight from the total sample weight and normalizing to the suture length. As shown in Figure 1A, the relative sample weight per millimeter did not differ significantly between the two materials. In contrast, the *Vicryl* samples were significantly longer than the *Monocryl* samples (Figure 1B; Wilcoxon rank-sum test, *p* = 0.016).

#### 3.2.2. Microbial Variance and Alpha Diversity

The microbial landscape detected on the sutures was not driven by the suture type, but rather by the patient origin of the suture material, as indicated by the largely distinct clusters in the PCoA (Figure 2). This suggests that inter-individual variability in the resident microbiota play a greater role in shaping the microbial composition than the suture type.

Microbial diversity was further characterized by assessing the Shannon and Simpson alpha diversity indices. According to the indices shown in Figure 3, the alpha diversity did not significantly differ between *Monocryl* and *Vicryl* (Shannon: *t*-test, *p* = 0.574; Simpson: Wilcoxon rank-sum test, *p* = 0.721).

#### 3.2.3. Material-Specific Differences in Bacterial and Host Cell Deposition

To quantify bacterial colonization of the two suture materials and to relate it to host-derived material deposition, we determined the 16S rRNA gene copy numbers as a proxy for the bacterial load and the albumin gene copy numbers as a marker of the host cell presence. Samples P25, P27, P34, and P52 were excluded from further analysis due to the absence of detectable 16S rRNA gene amplification in qPCR.

*Vicryl* sutures harbored significantly higher 16S rRNA gene copy numbers per millimeter compared to *Monocryl* sutures (Figure 4A; Wilcoxon rank-sum test, *p* ≤ 0.0001), indicating the increased bacterial colonization of *Vicryl* material. In contrast, the albumin gene copy numbers per millimeter were significantly higher in *Monocryl* sutures than in *Vicryl* sutures (Figure 4B), suggesting greater host cell deposition on *Monocryl*. Across all samples, the 16S rRNA gene copy numbers exceeded the albumin gene copy numbers by approximately 300-fold, consistent with a substantially higher abundance of bacterial than host-derived DNA on both suture types.

Taken together, these findings indicate material-specific differences in colonization patterns: *Vicryl* appears to favor bacterial accumulation, whereas *Monocryl* is associated with relatively greater host cell deposition.

#### 3.2.4. Microbial Composition: Relative Abundances

Both *Monocryl* and *Vicryl* sutures were colonized by a similar core set of genera, including *Bacteroides*, *Campylobacter*, *Capnocytophaga*, *Fusobacterium*, *Mycoplasma*, *Paraprevotella*, *Parvimonas*, *Porphyromonas*, *Prevotella*, *Rothia*, and *Staphylococcus* (Figure 5). Several genera were detected exclusively on *Monocryl* (*Dehalobacterium*, *Enterobacter*, *Filifactor*, *Haemophilus*, *Neisseria*, *Oribacterium*, *Streptococcus*, and *Veillonella*), whereas *Phocaeicola* was observed only on *Vicryl* sutures. However, these material-specific occurrences were not statistically significant. Given the limited sample size and pronounced inter-individual variability, these findings likely reflect patient-specific microbial differences rather than true material-dependent colonization patterns. Overall, no significant differences in relative microbial composition were detected between *Monocryl* and *Vicryl* sutures.

#### 3.2.5. Microbial Composition: Absolute Abundances

By means of the multiplication of the 16S rRNA gene copy numbers acquired by qPCR and relative abundances acquired through 16S rRNA sequencing, we were able to estimate the absolute abundance of the taxa (Figure 6). The samples P20, P44, P48, and P50 from the *Vicryl* cohort exhibited the highest bacterial load.

#### 3.2.6. Association of Suture Biomass with Host and Bacterial Load

To investigate whether an increased relative sample weight reflects a greater accumulation of host-derived material and/or bacterial colonization, we examined the relationship between the length-normalized relative sample weight and the molecular markers of host cells (albumin gene copy numbers) and the bacterial burden (16S rRNA gene copy numbers and ZOTU read counts).

Pearson’s correlation analyses were performed separately for *Vicryl* and *Monocryl* sutures using length-normalized parameters, including the relative sample weight (suture dry weight subtracted and normalized to the suture length), albumin gene copy numbers, 16S rRNA gene copy numbers, and ZOTU read counts. In *Vicryl* samples (Figure 7A), the relative sample weight was significantly positively correlated with the albumin gene copy numbers (ρ = 0.53, *p* = 0.0024). Moreover, the albumin gene copy numbers were significantly associated with ZOTU counts (ρ = 0.46, *p* = 0.00971), suggesting that an increased host material deposition coincided with a higher bacterial abundance. In *Monocryl* samples (Figure 7B), the relative sample weight demonstrated a strong positive correlation with the albumin gene copy numbers (ρ = 0.88, *p* = 9.54 × 10^−8^), indicating that the sample mass was predominantly driven by host-derived material. Moderate positive correlations were observed between the 16S rRNA gene copy numbers and relative sample weight (ρ = 0.42, *p* = 0.0492), as well as between the 16S rRNA gene copy numbers and albumin gene copy numbers (ρ = 0.46, *p* = 0.0308), pointing to a partial association between host cell accumulation and bacterial load.

Taken together, these findings suggest that an increased suture-associated biomass primarily reflects host cell deposition, particularly in *Monocryl* samples, while bacterial abundance appears to be linked to host material accumulation rather than independently driving the overall sample weight.

### 3.3. Secondary Outcomes

#### 3.3.1. Early Wound Healing Score

**Figure 8:**
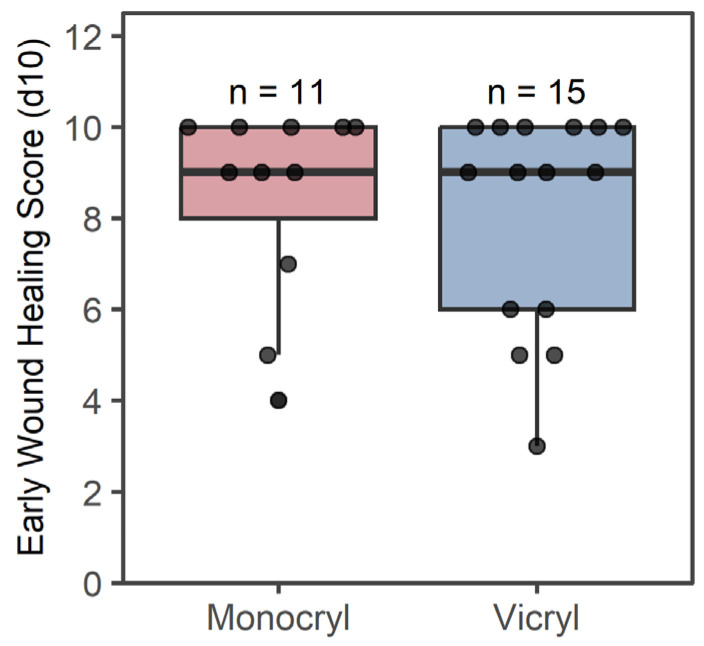
Mean EHS 10 days post-surgery for *Vicryl* and *Monocryl* sutures. Boxes show 1st and 3rd quartile and median and whiskers represent the 1.5x interquartile range.

Ten days post-surgery, 18 patients (8 in the *Monocryl* group and 10 in the *Vicryl* group) scored 9 or 10 points on the EHS, whereas 4 patients in the *Vicryl* group and 2 in the *Monocryl* group scored 5 to 7 points. The minimum EHS score on day 10 after surgery for both groups was seen in PT11 (*Vicryl*) with 3 points and in PT6 (*Monocryl*) with 4 points (as illustrated in Table 3 and Figure 8).

Moreover, no wound healing disorders or dehiscences were observed in either the *Monocryl* or *Vicryl* group across 10 days after the surgery. Consequently, no MRONJ occurred in the included patients over long-term observation (8 weeks).

#### 3.3.2. Mombelli Plaque and Bleeding Index Assessment (MPI, MBI)

As shown in Table 3, 14 patients received an MPI of 0 or 1 upon clinical assessment, whereas 2 patients (PT1 *Vicryl* and PT11 *Vicryl*) were categorized as MPI 2. Fifteen patients were categorized as MBI 0 or 1; however, one patient (PT21 *Monocryl*) reached Grade 3 of MBI.

#### 3.3.3. Smear of the Oral Cavity

Fifteen smears (with one smear being untraceable) were analyzed by the Institute of Clinical Microbiology, Immunology, and Hygiene of the University of Erlangen-Nürnberg. One patient (PT11 *Vicryl*) was colonized with *Staphylococcus aureus* and *Candida albicans*. PT5 *Vicryl* showed the colonization of *Candida glabrata* and *Enterobacter cloacae*. Two additional patients were positive for *C. albicans*. In the remaining patients, the microbiological cultures yielded components of a normal oropharyngeal microbiota.

## 4. Discussion

Postmenopausal osteoporosis is among the most common systemic skeletal diseases in older individuals and leads to increased fragility and fracture risk because of the reduced bone mass and loss of microarchitecture. Antiresorptive therapies, especially oral and intravenous bisphosphonates and denosumab, and, in some cases, also SERMs effectively reduce the risk of fracture and are therefore part of the standard treatment—despite the known, but overall rare, side effects such as MRONJ. In accordance with the AAOMS criteria, MRONJ is defined clinically. In the osteoporotic setting, the absolute risk of MRONJ is low. For oral bisphosphonates, it is typically in the range of ~0.02–0.1% (for treatment durations > 4 years up to ~0.21%), whereas, for denosumab, the risk is ~0.04–0.3%, but still much lower than in high-dose oncology scenarios. Moreover, dentoalveolar procedures—especially tooth extractions—are the most common triggers. Therefore, a careful pre/perioperative strategy with infection control and tension-free closure remains essential [33]. Prospective data from postmenopausal women also revealed that ongoing treatment with alendronate or zoledronate did not significantly impair mucosal (epithelial closure) and osseous healing (alveolar filling) after tooth extraction, provided that a standardized atraumatic protocol (including chlorhexidine rinses, antibiotics, and suture closure) was used. In the present study, the bone growth and the socket filling rate after 30/90 days were comparable between bisphosphonate-exposed and nonexposed patients; complete mucosal healing was regularly achieved [1]. This suggests that, with an adequate surgical technique, it is not necessary to interrupt antiresorptive therapy, provided that special care is taken during oral procedures in this patient group [1,34].

In this context, the question of local, influenceable factors in early wound healing—such as the suture technique and suture material—is more than theoretical: In an environment dominated by biofilms, the surface topography, capillarity, and retention tendency of the suture can contribute to the microbial load during wound healing. The evidence-based selection of a tension-free closure with the smallest possible bacterial footprint is therefore a plausible strategy for further reducing the risk of a severe complication such as MRONJ.

### 4.1. Primary Outcomes

A number of clinical studies have shown that, compared with monofilament synthetic sutures (e.g., poliglecaprone25/*Monocryl*, polypropylene, PTFE, and nylon), multifilament/braided sutures (e.g., Polyglactin 910/*Vicryl*) exhibited more bacterial colonization and retention niches [10,20]. A study of 10 materials revealed greater colonization on multifilaments and better healing scores for monofilaments [20]. A pilot study also revealed less biofilm on PTFE than on silk and significantly fewer bacteria adhered to PTFE than to silk in vivo [22,23]. The majority of studies therefore concluded that monofilaments perform better than multifilaments do, which is consistent with our results. However, a direct differential effect on wound healing has not been proven to date.

The quantification of absolute bacterial abundance demonstrated significantly higher 16S rRNA gene copy numbers on *Vicryl* sutures compared to *Monocryl*, indicating an increased bacterial colonization of the braided material. Four *Vicryl* suture samples exhibited a particularly high microbial load. The suture sample of PT5 *Vicryl* with EHS 5 was among them, although there was no reduction in EHS at the surgery sites of the remaining samples. The stronger association of microbiological abnormalities with low EHS values than with low plaque or bleeding indices is biologically plausible. Biofilms significantly impair early healing, and dysbiosis is associated with delayed wound healing. As the inter-individual signature of the oral microbiome varies in composition, it can at least partially overrule material effects [35,36,37].

Our results are consistent with studies that examined sutures from dentoalveolar surgeries performed on patients who were not diagnosed with osteoporosis or treated with bisphosphonates [9,10,11,12,13,14,15,16,17,18]. According to a review, monofilament sutures tend to harbor fewer bacteria. One clinical study by Sala-Perez et al. [11] revealed significantly lower numbers of bacteria on *Monocryl* Plus than on braided natural silk sutures after three days, and, clearly, fewer pathogens were detected. As *Monocryl* Plus is a suture made of poliglecaprone25 coated with antibacterial triclosan, fewer microorganisms are expected [10,11]. However, triclosan-coated *Vicryl* sutures resulted in the highest bacterial load [12], and, compared with uncoated *Vicryl* sutures, triclosan-coated *Vicryl* sutures resulted in the presence of up to 37% more bacteria and a greater number of pathogens among the adhered microorganisms [13]. Thus, the clinical advantage of the triclosan coating appears to be inconsistent and limited in duration (max. 72–96 h). For dentoalveolar procedures, the overall evidence favors monofilament material, and the coating can be considered an add-on, but it does not replace an atraumatic technique [19,21]. Two additional studies concluded that, compared with non-resorbable multifilament and monofilament sutures, bacteria are less adherent to monofilament resorbable sutures such as *Monocryl* [14,15]. In contrast, one study revealed that multifilament sutures (e.g., silk and *Vicryl* Plus) resulted in similar amounts of bacteria, regardless of whether they were resorbable or non-resorbable, and nylon was the only monofilament suture material that resulted in a lower bacterial load [16]. A study that compared four different suture materials revealed that monofilament sutures, although non-resorbable, exhibited the least microbial adherence [9]. Although various suture materials are used, monofilament sutures generally result in a lower bacterial load compared to multifilament sutures. Scanning electron microscopy demonstrated that microbial plaques predominantly adhered to the multifilament sutures, whereas monofilament sutures retained fewer bacteria due to their smoother surface structure [9,17]. This observation is consistent with our qPCR results showing higher 16S rRNA gene copy numbers on *Vicryl* compared to *Monocryl*. Finally, an in vitro study did not find significant differences in suture materials from *Ethicon* (e.g., *Vicryl* Plus and *Monocryl*) and revealed no significant differences among them in the adherence of microbes, except uncoated *Vicryl*, which had the highest bacterial counts [18].

The microbial profiles of *Monocryl* and *Vicryl* sutures were largely comparable in overall composition. The variance in the microbiota appeared to be driven primarily by the patient’s individual microbial signature rather than the suture material itself, as reflected by the patient-dependent clustering in the PCoA analysis. In particular, samples obtained in split-mouth designs, as well as repeated surgeries in the same patient, showed clear clustering patterns. This observation was further supported by the Shannon and Simpson diversity indices, which revealed no significant differences in alpha diversity between suture materials. These findings are consistent with previous reports demonstrating that host–microbe interactions and the individual oral microbiome are dominant determinants of the microbial community structure [37].

Although differences in the composition of suture-associated microbiota could theoretically arise either from the mere presence of a foreign body or from material-specific microbial affinities, the latter appears biologically plausible given the distinct structural and physicochemical properties of monofilament and multifilament sutures. Surface roughness, capillarity, and protein adsorption characteristics may influence the initial bacterial adhesion and subsequent biofilm development. However, despite the significantly higher absolute bacterial load on *Vicryl*, we did not observe material-dependent shifts in the relative community composition. Instead, inter-individual variability predominated. It is therefore likely that the potential material-specific affinities, if present, are subtle and were not detectable within the limited sample size and heterogeneous patient cohort of the present study.

With regard to the taxonomic composition, the majority of the detected genera corresponded to bacteria typically associated with a healthy oral microbiota. The physiological oral microbiome commonly includes genera such as *Campylobacter, Capnocytophaga, Rothia, Desulfobacter, Haemophilus, Veillonella,* and *Neisseria* [38]. In addition, *Oribacterium* [39] and *Mycoplasma salivarius* are regarded as commensal organisms and components of a normal oral microbial ecosystem [40].

Interestingly, the albumin gene copy numbers were significantly higher on *Monocryl* sutures, suggesting greater host cell deposition on the monofilament material. This finding suggests that host-derived material deposition does not simply parallel bacterial plaque accumulation and may instead be influenced by additional material properties. It is conceivable that differences in the surface chemistry, hydrophobicity, or protein adsorption characteristics of the monofilament material promote the retention of host proteins and cellular debris independent of bacterial biofilm formation. Correlation analyses further revealed that the relative sample weight was strongly associated with albumin gene copy numbers in both materials, particularly in *Monocryl*, where a very strong positive correlation was observed. Associations between the weight and bacterial load were comparatively weaker. These findings indicate that the overall suture-associated biomass is primarily driven by host-derived material rather than bacterial accumulation, while *Vicryl* appears to support higher bacterial colonization independent of the total sample weight.

The length of the collected suture samples differed significantly between materials, with *Vicryl* samples being longer than *Monocryl* samples. One possible explanation could be the difference in commercially available strand lengths (e.g., 45 cm for *Vicryl* versus 70 cm for *Monocryl*) [24], which may have influenced trimming and handling during placement and removal.

### 4.2. Secondary Outcomes

Owing to the oral microbiome, food detritus, and the subsequently formed biofilm, wound healing processes in the oral cavity are prone to be impeded by infections [9]. Dysbiosis of the oral microbiome can cause infections, e.g., periodontitis, and, when members of the microbiota adhere to the extraction site, delayed wound healing occurs [41].

In our study, the mean EHS increased over time, which resembled the physiological wound healing process that consists of inflammatory, proliferative, and remodeling phases. However, the different wound healing stages merge and cannot be considered separately. Inflammation already starts after a few hours, but can last for three [42] to seven [43] days after the procedure. The inflammatory phase is characterized by swelling, redness, and fibrin fibers, which lead to a lower EHS. Nonetheless, the majority of EHS scoring points (6) are awarded for the evaluation of the incision margins, categorized as either separate, in contact, or merged. This represents the crucial part of flap closure. In the context of MRONJ, the visible distance between incision margins is indicative of the risk of probeable and, consequently, exposed bone [44,45]. An earlier study demonstrated that wound healing following tooth extraction is prolonged in patients treated with bisphosphonates. In patients who had been receiving bisphosphonates for a period surpassing 5 years, it even exceeded six weeks without developing BRONJ [46]. The results of the present study demonstrated the healing during only the first ten days; however, these observations did not provide direct insights into the overall wound healing process. Since EHS is intended for the assessment of initial wound healing, which takes up to several weeks, it should be used only for observational purposes [42,43,44].

To judge the wound healing ability, the plaque and bleeding indices were determined individually for each patient on the day of surgery. These indices, however, did not seem to be relevant predictors for the healing process documented via the EHS in our study. A previous analysis of periodontal disease around implants revealed results similar to those of clinical indices (e.g., modified Mombelli Plaque Index), and the degree of inflammation of the probed tissue did not significantly correlate with wound healing [47]. In our study, two patients had a higher plaque index, and one patient had a higher bleeding index. The EHS was low (3 points) for the patient (PT11 *Vicryl*) who had an MPI of 2 compared to the other patients (Table 3).

Furthermore, the EHS was observed to be low in patients who exhibited microbiological anomalies via smear tests as opposed to a higher plaque or bleeding index. The patient mentioned above (PT11 *Vicryl*) was colonized with *S*. *aureus* and *C*. *albicans*. The patient diagnosed with colonization by *C. glabrata* and *E. cloacae* (PT5 *Vicryl*) had an EHS of 5. Two further patients were found to be positive for *C. albicans*, one patient who was part of the split-mouth design (with EHS 5 using *Vicryl,* and EHS 4 and EHS 5 using *Monocryl*), as well as another *Vicryl* patient with EHS 10. The remaining patients, who showed normal preoperative smear results, reached 9–10 points on the EHS, except for two *Vicryl* patients, both with EHS 6, and one *Monocryl* patient with EHS 7. The suture samples obtained from the extraction wounds mentioned above were not observed to have higher bacterial loads, except for one *Vicryl* patient. The observation that the plaque and bleeding indices correlate only to a limited extent with the EHS in the first few days is consistent with the purpose of the EHS. It primarily records early re-epithelialization and has been validated for the initial phase after periodontal surgery; thus, its significance for later healing phases is naturally limited [44].

However, the bacteria found on the suture samples did not correspond to the microorganisms detected on the swabs. The extraction sites that attained an EHS ≤ 7 were partly colonized with genera that included species that are associated with periodontitis and severe oral bone loss in osteoporotic women. These species include *Porphyromonas gingivalis, Prevotella intermedia*, *Campylobacter rectus, Capnocytophaga* sp., and *Fusobacterium nucleatum* [48,49]. There are descriptions of cohorts of postmenopausal women in which such species were associated with alveolar bone loss. In particular, *Fusobacterium nucleatum* acts as a bridge organism that promotes the establishment of *P. gingivalis* and other pathobionts. This mechanism therefore seems plausible as to why individual microbial profiles can impair early wound closure despite clinically similar indices [48,50,51]. However, it is important to note that the present study does not allow us to answer the question whether the bacterial species colonizing the suture samples influenced the process of wound healing. Furthermore, the genera of the species mentioned above were detected in other samples that did not show a reduced EHS.

In contrast to other studies, which obtained evidence that wound closure with monofilament synthetic suture material caused fewer tissue reactions and better wound healing outcomes compared to multifilament or natural materials [9,10], our results demonstrated that there were no significant differences in the EHS between *Monocryl* and *Vicryl*. The overall mean EHS was above 8, and most of the patients scored 9 to 10 points at 10 days post-surgery, indicating an effective standardized surgical protocol. Nevertheless, follow-up examinations three weeks post-surgery would provide more insight into the definitive wound healing outcome.

On this basis, it appears that the choice of material influences the bacterial load, whereas the EHS under standardized protocols often depends more on surgical technique and patient variability [37].

There are several limitations of the study that need to be mentioned and discussed. The present study should be interpreted as an exploratory analysis. Given the retrospective registration and the absence of a fully predefined prospective protocol, the findings are hypothesis-generating rather than confirmatory and should be interpreted with appropriate caution. To achieve an accurate interpretation of the estimated absolute abundance of the bacterial burden as determined by qPCR, it is essential to acknowledge the potential variability in the number of copies of the 16S rRNA gene present within individual bacterial taxa. This variability, which can range from 1 to 15 copies [52], was not considered in the present study. Corrections using the ribosomal RNA operon copy number database (rrnDB) and model-based copy number compensation could lead to improvements in the future. Furthermore, the small number of patients included in this study should be mentioned; a larger number of patients were not included because the methodological approach was established internally. However, this small number of patients certainly reduced the strength of the evidence. Finally, it should be noted that multiple samples were obtained from the same patient, which may have introduced a non-independence of the observations and should be considered when interpreting the results. The relationship between the suture material, bacterial adhesion, and MRONJ risk remains indirect and speculative, as MRONJ was not an endpoint of this study and no cases occurred during follow-up; therefore, the results should not be interpreted as definitive evidence for the clinical superiority of one suture material but rather as exploratory findings requiring confirmation in future prospective trials.

Although the study was registered (DRKS00037740) as non-interventional, it included the controlled allocation of suture materials and split-mouth elements, which are more consistent with an interventional design. This discrepancy is due to the retrospective registration and simplified categorization in the registry. In earlier versions of the manuscript, the study design was described as a prospective cohort study. This terminology has been revised to more accurately reflect the actual study conduct. The registry has not been modified post hoc, and all deviations from the registered study description are transparently disclosed in the present manuscript.

## 5. Conclusions

Compared with *Monocryl*, the multifilament suture material *Vicryl* exhibited significantly higher 16S rRNA gene copy numbers, indicating an increased bacterial colonization. In contrast, *Monocryl* showed significantly higher albumin gene copy numbers, suggesting greater host-derived material deposition on the monofilament surface. Correlation analyses further demonstrated that the suture-associated biomass was primarily driven by host cell accumulation, particularly in *Monocryl*, whereas associations between the weight and bacterial load were less pronounced. Despite these material-dependent differences in bacterial and host DNA abundance, no distinct microbial community profile was observed between *Vicryl* and *Monocryl* sutures. Moreover, the clinical healing outcomes according to the EHS remained comparable, even in the presence of significantly different bacterial loads. Therefore, within the limitations of this study, including the relatively small sample size, there is no clear clinical rationale to favor one suture material over the other. To minimize the risk of MRONJ, emphasis should instead be placed on adherence to an appropriate surgical protocol. In the present study, this included the use of a rotational flap with mesial relief incision, the careful rounding of bone edges, and perioperative antibiotic therapy.

## Figures and Tables

**Table 1:** General patient data. Patient ID, the antiresorptive agent and the type, frequency, and time span of its intake, and the suture material (V = *Vicryl*, M = *Monocryl*), as well as the surgical area of the extraction, are listed. Patients with a split-mouth design or two surgeries are marked (*). NA indicates not available data.

Patient ID	Antiresorptive Agent	Type (i.v./p.o.)/ Frequency of Intake	Time Span of Intake	Material V/M	Surgical Area (Region)
PT1	Alendronate	p.o., weekly	To date	V	32–42
PT2	Risedronate	p.o., weekly	To date	V	45
PT3	Alendronate	NA	To date	V	45
PT5	Alendronate	p.o., weekly	2021–2023	V	27
PT6 *	Risedronate	p.o., weekly	To date	V M M	26–27 44–45 46–47
PT7	Zolendronate	i.v., 1x/year	To date	V	37
PT8 *	Alendronate	NA	To date	V M	14 25
PT9 *	Ibandronate	NA	To date	V M	43, 33–34 12
PT10	Alendronate Denosumab	NA NA	2014–2016 2019–to date	V	38
PT11	Alendronate	p.o., weekly	To date	V	46
PT12	Risedronate	NA	To date	V	15
PT13 *	NA	i.v., 4x/year	Until 2023	V M	Lower jaw Upper jaw
PT14	NA Risedronate Strontiumranelat Alendronate Zoledronate	NA NA NA NA NA	2002–2006 2007 2009–2010 2010–2014 2014–2016	V	26
PT15 *	Alendronate Denosumab	p.o., daily i.v., 2x/year	Until 2022 To date	V M	15 34
PT16	Risedronate	p.o., weekly	2005–2008	V	15–17
PT17	Denosumab	i.v., monthly	To date	M	12
PT18	Alendronate	p.o., weekly	To date	M	45–47
PT19	Ibandronate	i.v., 4x/year	To date	M	NA
PT20	Alendronate	NA	Discontinued	M	34
PT21	Alendronate	NA	To date	M	22

**Table 2:** Primary outcomes, used for computational and statistical analysis. V = *Vicryl*, M = *Monocryl*.

Suture ID	16S Copy Number	Albumin Copy Number	Weight [mg]	Length [mm]	Material (V/M)	Patient ID	Surgical Area (Region)
P1	1.99 × 10^7^	6.19 × 10^3^	1.87	7	M	PT18	45–47
P2	2.01 × 10^8^	2.72 × 10^5^	6.58	12	V	PT5	27
P4	2.00 × 10^8^	2.19 × 10^5^	7.27	10	V	PT1	32–42
P5	1.67 × 10^7^	5.01 × 10^5^	4.75	12	V	PT6	26–27
P6	1.14 × 10^8^	3.90 × 10^4^	3.76	10	V	PT2	45
P7	1.55 × 10^7^	3.74 × 10^4^	4.23	6	M	PT15	34
P8	9.22 × 10^7^	1.31 × 10^4^	3.41	18	M	PT19	NA
P9	1.36 × 10^6^	2.44 × 10^5^	4.24	9	M	PT6	46–47
P10	4.44 × 10^6^	7.62 × 10^3^	2.09	5	M	PT17	12
P11	7.92 × 10^7^	7.08 × 10^3^	3.49	9	M	PT17	12
P12	1.72 × 10^8^	1.47 × 10^5^	9.92	9	V	PT1	32–42
P14	1.50 × 10^8^	1.52 × 10^5^	9.09	11	V	PT3	45
P15	4.44 × 10^7^	5.25 × 10^5^	5.94	12	V	PT2	45
P16	4.79 × 10^6^	4.46 × 10^5^	3.51	10	M	PT6	46–47
P17	4.86 × 10^6^	5.63 × 10^5^	5.64	16	V	PT6	26–27
P18	2.11 × 10^7^	5.64 × 10^3^	4.48	8	M	PT18	45–47
P19	7.84 × 10^6^	8.09 × 10^3^	2.21	14	M	PT15	34
P20	6.57 × 10^8^	6.40 × 10^5^	8.39	10	V	PT5	27
P21	7.38 × 10^7^	2.26 × 10^5^	2.76	8	V	PT3	45
P22	5.78 × 10^5^	1.24 × 10^5^	1.93	12	M	PT19	NA
P23	6.59 × 10^6^	3.54 × 10^4^	2.66	9	M	PT20	34
P24	2.05 × 10^7^	5.28 × 10^3^	4.18	8	M	PT20	34
P25	NA	1.31 × 10^5^	3.28	9	M	PT6	44–45
P26	2.68 × 10^4^	7.48 × 10^4^	2.35	22	M	PT6	44–45
P27	NA	9.56 × 10^5^	7.43	14	V	PT7	37
P28	1.23 × 10^7^	2.39 × 10^5^	2.56	13	V	PT7	37
P29	1.78 × 10^7^	9.17 × 10^5^	4.81	10	V	PT8	14
P30	8.01 × 10^5^	3.44 × 10^5^	3.44	13	M	PT8	25
P31	2.76 × 10^6^	3.50 × 10^5^	3.83	21	M	PT8	25
P32	1.36 × 10^8^	4.29 × 10^5^	2.2	12	V	PT8	14
P33	1.02 × 10^8^	1.16 × 10^5^	5.56	18	V	PT9	43, 33–34
P34	NA	5.42 × 10^4^	4.46	10	V	PT9	43, 33–34
P35	5.26 × 10^6^	1.81 × 10^4^	3.41	7	M	PT9	12
P36	8.42 × 10^6^	1.35 × 10^5^	2.9	12	M	PT9	12
P37	1.97 × 10^8^	2.33 × 10^5^	6.45	27	V	PT10	38
P38	5.70 × 10^7^	1.13 × 10^5^	3.23	15	V	PT10	38
P39	1.04 × 10^7^	2.92 × 10^5^	2.63	11	V	PT11	46
P40	7.56 × 10^6^	3.36 × 10^5^	4.06	12	V	PT11	46
P41	9.58 × 10^6^	2.55 × 10^4^	3.24	11	M	PT21	22
P42	1.19 × 10^7^	6.84 × 10^4^	3.08	10	M	PT21	22
P43	1.16 × 10^8^	2.06 × 10^5^	2.88	8	V	PT12	15
P44	6.36 × 10^8^	2.69 × 10^5^	7.65	26	V	PT12	15
P45	1.56 × 10^7^	2.94 × 10^5^	1.57	17	M	PT13	Upper jaw
P46	2.20 × 10^7^	1.53 × 10^4^	6.02	5	M	PT13	Upper jaw
P47	4.67 × 10^7^	2.83 × 10^5^	4.89	15	V	PT13	Lower jaw
P48	6.08 × 10^8^	8.79 × 10^4^	6.41	16	V	PT13	Lower jaw
P49	2.22 × 10^8^	2.26 × 10^5^	3.75	15	V	PT14	26
P50	3.79 × 10^8^	4.38 × 10^5^	7.33	35	V	PT14	26
P51	2.21 × 10^7^	7.07 × 10^5^	3.07	10	V	PT15	15
P52	NA	3.92 × 10^5^	3.44	23	V	PT15	15
P53	1.38 × 10^8^	2.66 × 10^5^	1.22	13	V	PT16	15–17
P54	1.67 × 10^8^	3.12 × 10^5^	1.84	14	V	PT16	15–17

**Table 3:** Overview of secondary outcomes listed by Patient ID. V = Vicryl, M = Monocryl.

Patient ID	Material V/M	EHS d10	MPI	MBI
PT1	V	10	2	1
PT2	V	10	1	1
PT3	V	9	1	1
PT5	V	5	1	0
PT6	V M M	5 4 5	1 1 1	1 1 1
PT7	V	6	1	1
PT8	V M	9 9	1 1	1 1
PT9	V M	10 10	1 1	1 1
PT10	V	10	1	1
PT11	V	3	2	1
PT12	V	10	1	1
PT13	V M	9 9	00	00
PT14	V	9	1	1
PT15	V M	6 10	1 1	1 1
PT16	V	10	1	1
PT17	M	10	1	1
PT18	M	10	1	1
PT19	M	10	1	1
PT20	M	7	1	0
PT21	M	9	1	3

## Data Availability

The datasets used and/or analyzed during the current study are available from the corresponding author upon reasonable request.

## Abbreviations

The following abbreviations are used in this manuscript:

AAOMS	American Association of Oral and Maxillofacial Surgeons
ARONJ	Antiresorptive-related osteonecrosis of the jaw
BRONJ	Bisphosphonate-related osteonecrosis of the jaw
CSH	Clinical signs of hemostasis
CSI	Clinical signs of inflammation
CSR	Clinical signs of re-epitheliazation
DNA	Desoxyribonucleic Acid
EHS	Early Wound Healing Score
HIV	Human Immunodeficiency Virus
M	*Monocryl*
MBI	Mombelli Bleeding Index Assessment
MPI	Mombelli Plaque Index Assessment
MRONJ	Medication-related osteonecrosis of the jaw
ONJ	Osteonecrosis of the jaw
PCoA	Principal Coordinate Analysis
PTFE	Polytetrafluoroethylene
qPCR	Quantitative Polymerase Chain Reaction
RANKL	receptor activator of nuclear factor κ-B ligand
rRNA	Ribosomal ribonucleic acid
SERMs	selective estrogen receptor modulators
V	*Vicryl*
ZOTU	Zero-radius Operational Taxonomic Unit
